# Retrotransposons in Bone and Joint Diseases

**DOI:** 10.1096/fba.2026-00044

**Published:** 2026-05-18

**Authors:** Ye Liu, H. Josh Jang, Tao Yang

**Affiliations:** ^1^ Department of Cell Biology Van Andel Institute Grand Rapids Michigan USA

**Keywords:** epigenetic, osteoarthritis, osteoporosis, retrotransposon

## Abstract

Retrotransposons, including non‐LTR elements such as LINEs and viral‐derived endogenous retroviruses (ERVs), have long been dismissed as “junk DNA” and thought to be biochemically inert. However, emerging evidence suggests that the erosion of epigenetic control during aging and pathological states can lead to the awakening of these dormant genetic elements. Although the role of ERVs in cancer and neurodegeneration is increasingly recognized, their impact on musculoskeletal health has received little attention. This perspective review synthesizes recent and previous findings linking retrotransposon reactivation (particularly ERVs) to osteoarthritis (OA), rheumatoid arthritis (RA), and potentially osteoporosis. We discuss the epigenetic mechanisms that typically silence ERVs in musculoskeletal tissues, how these mechanisms fail in disease, and how the resulting reactivation leads to viral and molecular mimicry. These processes trigger both innate and adaptive immune responses, as well as cellular senescence. Finally, we highlight the therapeutic potential of targeting retrotransposon dysregulation, including its encoded proteins and nucleic acid‐sensing pathways to treat chronic bone and joint disorders.

## Graphical abstract

This graphical abstract illustrates how epigenetic erosion during aging and disease reactivates retrotransposons, particularly endogenous retroviruses (ERVs), in musculoskeletal tissues. Their activation induces viral and molecular mimicry, triggering innate and adaptive immune responses and promoting cellular senescence. These processes contribute to the pathogenesis of osteoarthritis, rheumatoid arthritis, and potentially osteoporosis. Targeting retrotransposon activity and nucleic acid‐sensing pathways may offer new therapeutic strategies for bone and joint diseases.

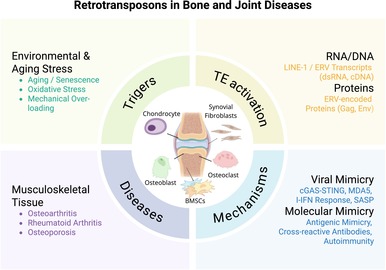

## 1 Introduction

Retrotransposons constitute a major class of transposable elements (TEs) in vertebrate genomes and include both long terminal repeat (LTR)–containing elements, such as endogenous retroviruses (ERVs), and non‐LTR elements, such as long interspersed nuclear elements (LINEs). Endogenous retroviruses (ERVs) are remnants of ancient retroviral infections that have become permanently integrated into the germline genomes of vertebrates [1]. These sequences, once exogenous infectious agents, are now vertically inherited and collectively comprise approximately 8%–10% of the human genome [2, 3]. Structurally, ERVs retain canonical retroviral features, including internal *gag*, *pol*, and *env* genes flanked by long terminal repeats (LTRs), which harbor abundant transcription factor binding sites and exhibit robust cis‐regulatory potential. Importantly, the majority of the ERVs have accumulated mutations that disrupt canonical regulatory programs or generate new gene‐regulatory logic, and many now persist as solitary LTRs as a result of recombination events over evolutionary time. In addition to ERVs, LINEs represent another major class of retrotransposons, constituting a substantial and biologically active fraction of the mammalian genome. LINEs are autonomous non‐LTR elements that mobilize via an RNA intermediate and primarily affect genomic stability. Despite these differences, both ERVs and LINEs depend on stringent epigenetic repression to maintain genomic integrity.

To limit retrotransposon activity and preserve genomic stability, host cells have evolved robust epigenetic mechanisms to suppress their transcription. Across both developmental and adult tissues, retrotransposon activity is primarily controlled by DNA methylation, trimethylation of histone H3 at lysine 9 (H3K9me3), and histone deacetylation. These repressive pathways are coordinated by epigenetic enzymes including SETDB1, TRIM28/KRAB‐ZFP complexes, and DNA methyltransferases (DNMTs) [1]. However, accumulating evidence highlights the complexity and tissue‐specificity of particular epigenetic suppression mechanisms that regulate ERV expression. For example, the conditional deletion of the histone methyltransferase *Setdb1*, which deposits H3K9me3, in mouse embryonic endoderm results in the derepression of the intracisternal A particle (IAP) family of ERVs in visceral endoderm (VE) but not in definitive endoderm (DE). Conversely, deletion of *Dnmt1* leads to derepression of IAP elements in both lineages, even when H3K9me3 marks remain intact [4]. These findings also suggest that while DNA methylation often plays a dominant role in maintaining ERV silencing, H3K9me3 serves as a compensatory or critical stabilizer during development. Importantly, these regulatory barriers are not immutable as they can become compromised under pathological or aging‐associated conditions, resulting in so‐called “epigenetic erosion.” Aberrant reactivation or derepression of ERVs has consequently been implicated in an expanding range of human diseases. In cancer and autoimmunity, ERV reactivation contributes to pathogenesis through two distinct but interrelated mechanisms, viral mimicry and molecular mimicry [5, 6, 7]. In viral mimicry, ERV‐derived nucleic acids, such as double‐stranded RNA (dsRNA) or cytoplasmic DNA, activate innate immune sensors including RIG‐I‐like receptors, Toll‐like receptors (TLRs), and the cGAS‐STING axis [8, 9, 10]. In contrast, molecular mimicry occurs when ERV‐encoded proteins act as antigens that share structural similarities with host self‐proteins, potentially inducing cross‐reactive autoantibodies and adaptive immune dysregulation [6, 11, 12]. For example, activation of human ERV‐K (HERV‐K), one of the most recently integrated and transcriptionally competent ERV families in human genome, has been shown to drive inflammatory signaling in senescent cells, linking ERVs directly to aging‐related pathologies [12].

Age‐related musculoskeletal pathologies, including osteoarthritis (OA), rheumatoid arthritis (RA), and osteoporosis, are characterized by chronic inflammation, dynamic tissue remodeling, and heightened cellular senescence. These pathological hallmarks closely parallel biological contexts in which ERVs have been shown to exert functional roles in other organ systems [13, 14, 15]. However, the contribution of retrotransposons to age‐related bone and joint diseases remains largely unexplored. For example, a previous study detected differential ERV‐3 mRNA expression in peripheral blood mononuclear cells (PBMCs) from patients with OA compared to healthy controls [16], suggesting that specific ERV activity may be associated with the pathophysiology of OA. Notably, emerging evidence also implicates other classes of retrotransposons in inflammatory and degenerative processes, although their roles in musculoskeletal tissues remain largely unexplored. Collectively, these observations point to a potential paradigm shift in musculoskeletal research, positioning retrotransposon dysregulation not merely as reactivation of dormant genetic remnants, but as active contributors and potential therapeutic targets in the chronic inflammation and cellular senescence that define bone and joint pathologies.

## 2 Retrotransposons Expression and Regulation in Musculoskeletal Tissues

To understand how retrotransposons, particularly ERVs, contribute to skeletal disease, it is essential to first delineate how they are maintained in a transcriptionally silent state across the diverse cell types that constitute the bone and joint microenvironment. The skeletal system comprises cells of distinct lineages and lifespans, including short‐lived but highly active osteoclasts (of the hematopoietic lineage), mesenchymal‐derived bone‐forming osteoblasts, and their long‐lived, matrix‐embedded osteocytes and chondrocytes. Each lineage faces unique epigenetic challenges in maintaining the “dormant genome” in check.

In healthy musculoskeletal tissues, ERVs are maintained in a heterochromatic state, primarily through H3K9me3 and DNA methylation. Osteoclasts originate from the immune system; their precursors, bone marrow monocytes (BMMs), are inherently poised to sense pathogen‐associated molecular patterns (PAMPs) [17]. During aging, the epigenetic suppression of ERVs may erode in these cells, thereby compromising their capacity to differentiate into functional bone‐resorbing osteoclasts. Instead, these cells may become arrested in an inflammatory state driven by aberrant interferon signaling [18, 19]. Conversely, chondrocytes and osteocytes reside within the skeletal tissue matrix for decades. These long‐lived cell types are particularly prone to epigenetic drift—the gradual accumulation of epigenetic errors over time. As the fidelity of DNA methylation and H3K9me3 maintenance wanes throughout aging or acute injury, these cells may become chronic sources of retrotransposon‐derived antigens, contributing to sterile inflammation via a viral mimicry response [12].

**FIGURE 1:**
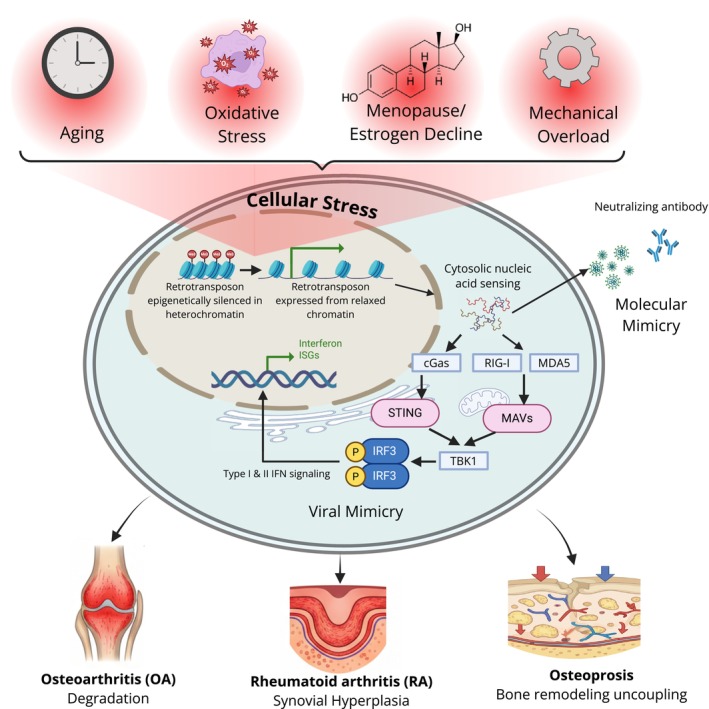
Mechanisms of retrotransposon activation in musculoskeletal pathologies. External cellular stressors, including aging, oxidative stress, mechanical loading, and estrogen decline (menopause), disrupt the homeostatic epigenetic repression of retrotransposons, allowing for the aberrant reactivation of ERV‐ or LINE‐1‐derived products, including nucleic acids and encoded proteins. These elements trigger dual pathogenic pathways: (1) Viral mimicry, in which cytosolic nucleic acids are sensed by pattern‐recognition receptors (PRRs) such as cGAS, MDA5, and RIG‐I, establishing a state of chronic interferon signaling and sterile inflammation; and (2) Molecular mimicry, in which retrotransposon‐derived proteins (e.g., HERV‐K Env) act as antigens that trigger cross‐reactive adaptive immune responses and autoantibody production. These persistent inflammatory and autoimmune environments drive tissue‐specific pathologies, including cartilage degradation in OA, autoimmunity and synovial hyperplasia in RA, and the uncoupling of bone remodeling in osteoporosis.

This viral mimicry is primarily driven by ERV reactivation in skeletal tissue, which produces cytosolic nucleic acids recognized by pattern‐recognition receptors (PRRs), such as RIG‐I/MDA5 (for RNA) and cGAS (for DNA). These molecular sensors trigger the phosphorylation of IRF3/7 and NF‐κB, leading to the secretion of Type I interferons (IFN‐α/β) and pro‐inflammatory cytokines (IL‐6 and TNF‐α) (Figure 1) [20, 21]. Although viral mimicry can theoretically arise from multiple retrotransposon classes, including LINE‐1, it is most robustly linked to ERV activation due to their retroviral origin and propensity to generate immunostimulatory nucleic acids. In joint tissues, in the context of bone, this response occurs both within resident cells, such as osteoclast precursors, osteocytes, osteoblasts, and chondrocytes, and by recruiting innate and adaptive immune cells, including macrophages, dendritic cells, and T cells. The resulting cytokines amplify local inflammation, disrupt osteoblast–osteoclast coupling, and reinforce cellular senescence, thereby contributing to age‐related bone and joint pathologies. In osteoclasts, acute, cell‐intrinsic IFN signaling acts as a protective brake to limit bone resorption, whereas chronic, low‐level IFN signaling driven by constitutive ERV activation represents a distinct pathological state. Furthermore, evidence from other model systems suggests that retrotransposon activation triggers innate immune sensing pathways, which promote a Senescence‐Associated Secretory Phenotype (SASP) [22]. In the context of the joint, such paracrine signaling may allow damaged cells to influence neighboring tissues, thereby accelerating cartilage degeneration and bone loss [23, 24, 25, 26].

## 3 Retrotransposons in Osteoarthritis

OA has long been considered a “wear‐and‐tear” disease, driven primarily by mechanical stress and joint overuse [27]. Recent breakthroughs, however, have highlighted the role of retrotransposon activation and age‐related molecular and immune dysregulation in driving joint degeneration: related joint degeneration [12]. For example, the murine ERV, *MMTV*, is significantly upregulated in the articular cartilage of aged mice [12]. This ERV reactivation correlated strongly with hallmark features of OA, including cartilage thinning, loss of proteoglycans, and reduced Ki67‐positive chondrocytes. Transcriptional inhibition of *MMTV* via intra‐articular CRISPR interference reversed degenerative changes, leading to increased cartilage thickness, improved bone density, and enhanced grip strength, supporting a causal role for ERV activation in OA pathogenesis rather than a passive bystander effect. Furthermore, systemic or local treatment with the nucleoside reverse transcriptase inhibitor Abacavir restored healthy tissue function. Abacavir‐treated aged mice exhibited reduced cellular senescence and inflammation in joint tissues, suggesting that blocking ERV transcription can mitigate age‐associated tissue degeneration.

Mechanistically, these retrotransposon‐derived transcripts appear to be reactivated by the erosion of epigenetic control. Aligning with the murine findings, our group has identified a specific epigenetic signature in human OA pathology that associates with ERV dysregulation. We observed significantly increased chromatin accessibility at specific ERV loci compared to healthy controls by profiling the chromatin landscape of human OA cartilage [28]. This work further suggests that epigenetic derepression, likely due to the loss of heterochromatic marks such as H3K9me3, precedes and primes ERV transcriptional activation.

## 4 Retrotransposons in Rheumatoid Arthritis

Unlike OA, rheumatoid arthritis (RA) is a systemic autoimmune disease characterized by chronic inflammation and aberrant activation of synovial fibroblasts. Aberrant activation of the cGAS‐STING pathway and type I IFN signaling is now recognized as a hallmark of RA pathology [29]. Seminal work examining the genomic instability of RA synovial fibroblasts (RASFs) revealed a striking upregulation of Long Interspersed Nuclear Elements (LINE‐1), another class of retrotransposons, alongside ERV‐related sequences such as ERV‐E and ERV‐HC2 [30]. Importantly, LINE‐1 and ERV reactivation is spatially restricted to specific regions within the joint, particularly at sites of active cartilage and bone erosion, suggesting a direct contribution to local tissue destruction. Notably, this expression pattern appears to be disease‐specific, as these elements were largely undetectable in OA control tissues in this study, highlighting distinct retrotransposon activity between degenerative and autoimmune pathologies.

The reactivation of LINE‐1 s and ERVs in RASFs has been shown to be closely linked to epigenetic erosion. RASFs are known to exhibit a globally hypomethylated genome [30]. Consistent with this observation, treatment of healthy synovial fibroblasts with the DNA demethylating agent 5‐aza‐2′‐deoxycytidine is sufficient to induce LINE‐1 expression, thereby recapitulating the RA phenotype. This suggests that the loss of repressive DNA methylation is a primary driver of retrotransposon reactivation in RA. More previous studies have corroborated this retroviral burden, identifying elevated levels of HERV‐K (HML‐2) transcripts in the synovial tissue and peripheral blood of RA patients, which correlate with disease activity and inflammatory cytokine levels [11, 31].

The functional consequences of this LINE‐1 reactivation are substantial. LINE‐1 activation alters the transcriptomic landscape, upregulating pro‐inflammatory stress kinases, such as SAPK4/p38 isoforms, and fibrosis markers such as galectin‐3 binding protein [30]. In addition, the accumulation of LINE‐1‐derived cytosolic nucleic acids can act as endogenous danger signals, engaging innate immune sensors including MDA5 or cGAS, and thereby triggering type I IFN response [32]. This form of viral mimicry creates a self‐perpetuating inflammatory loop in which epigenetic erosion drives ERV and LINE‐1 expression, and the ensuing innate immune activation promotes further tissue damage and autoimmunity, potentially explaining the chronic nature of RA synovitis.

## 5 Retrotransposons in Osteoporosis

Osteoporosis is a systemic skeletal disease characterized by low bone mass and microarchitectural deterioration of bone tissue, leading to enhanced bone fragility and a consequent increase in fracture risk [33]. At the cellular level, osteoporosis arises from an uncoupling of the bone remodeling process, where osteoclast‐mediated bone resorption outpaces osteoblast‐mediated bone formation. Beyond hormonal changes and aging, chronic sterile inflammation plays a central role in osteoimmunology by disrupting bone homeostasis [19, 34]. Given the capacity of LINE‐1 s and ERVs to drive inflammatory signaling, dysregulation of these retrotransposons in osteoporosis represents a compelling yet understudied axis in the pathogenesis of osteoporotic bone loss.

Syncytin‐1 (HERV‐W), best characterized for its role in mediating placental fusion, is also expressed in differentiating osteoclasts and is required for the fusion of mononuclear precursors into functional, multinucleated bone‐resorbing cells [35]. Furthermore, a recent study demonstrated that transient LINE‐1 transcription promotes osteoblast mineralization during fracture repair via the PKR sensing pathway [7], suggesting that controlled retrotransposon activity is integral to bone remodeling. However, the tight regulation of these elements is likely disrupted during aging or estrogen deficiency. Loss of estrogen is associated with increased oxidative stress and elevated levels of pro‐inflammatory cytokines (IL‐6 and TNF‐α), conditions known to erode epigenetic silencing marks. In this context, unchecked ERV reactivation represents a compelling mechanistic link in post‐menopausal osteoporosis, as activation of the cGAS‐STING/NF‐κB axis provides a biological explanation for the sustained inflammatory signaling that drives bone loss [36, 37].

The potential involvement of ERV dysregulation in bone loss introduces a complex biological paradox regarding interferon signaling. Classically, type I IFN (IFN‐β) has been defined as a negative regulator of osteoclastogenesis. A previous study demonstrated that RANKL signaling induces *Ifnb1* expression as an autoregulatory “brake” to prevent excessive bone resorption [18]. However, this classical model describes an acute, self‐limiting feedback loop. In contrast, ERV reactivation represents a state of chronic, low‐grade viral mimicry accompanied by sustained interferon expression. We postulate that constitutive exposure to ERV‐derived transcripts drives prolonged interferon activation within the tissue microenvironment, fundamentally distinct from the transient physiological response observed during acute inflammation. Under this proposed model, chronic IFN signaling in the bone marrow microenvironment could potentially induce a senescence‐like phenotype in osteoprogenitors or exhaustion of the stem cell pool, a hypothesis that would explain the disruption in bone remodeling. Elucidating the mechanisms by which ERV epigenetic regulation converts IFN signaling from a protective to a destructive program remains a critical area for future investigation.

## 6 Shared Mechanisms and Hypotheses Across Diseases

Despite their clinical differences, OA, RA, and osteoporosis converge on age‐related epigenetic dysregulation and inflammation. Genome‐wide association studies (GWAS) have identified numerous genomic loci associated with these musculoskeletal disorders. However, the presence of a risk variant does not guarantee disease progression [38]. Most disease‐associated SNPs reside in non‐coding regulatory regions, implying that they are not inherently functional or transcriptionally active [39, 40]. Instead, their effects on gene expression and disease risk are cell type‐ and context‐dependent, requiring an appropriate epigenetic landscape to become functionally engaged. Notably, many of these non‐coding regulatory elements, such as enhancers and promoters, are originally derived from retrotransposons [41, 42]. We propose a “risk variant–epigenetic erosion–retrotransposon activation” interaction model to explain how genetic predisposition translates into late‐onset musculoskeletal disease. It is increasingly recognized that aging and chronic environmental stress erode heterochromatic repression (e.g., loss of DNA methylation or histone H3K9me3) [43, 44]. In the musculoskeletal system, this epigenetic landscape is highly dynamic, specifically, DNA methylation patterns and their regulators, such as Ten‐eight translocation (TET) enzymes, play indispensable roles in skeletal development, fracture repair, and the maintenance of joint homeostasis [45, 46, 47, 48, 49, 50]. This loss of epigenetic fidelity not only unlocks genetic risk variants but also allows dormant retrotransposons to become transcriptionally active. Once reactivated, ERV‐derived nucleic acids trigger innate immune sensing pathways, establishing persistent sterile inflammation that promotes tissue degeneration and aggravates bone remodeling.

Recognizing ERVs as active drivers of disease opens a new frontier for clinical intervention. The most immediate translational opportunity lies in Nucleoside Reverse Transcriptase Inhibitors (NRTIs), such as Abacavir or Lamivudine. Originally designed for HIV, these drugs have shown efficacy in ameliorating OA and bone loss in preclinical models by blocking the reverse transcription of ERV RNA into immunogenic cDNA [22, 51]. Currently, joint diseases are often diagnosed only after structural damage has occurred [52]. Specific ERV expression profiles in blood or synovial fluid could serve as sensitive, early biomarkers of “epigenetic health,” flagging patients at risk of rapid progression before radiographic changes appear. Despite this promise, critical gaps remain. Future research should prioritize the characterization of the specific retrotransposon families that are reactivated in human bone diseases and dissecting the precise causality between viral mimicry and cellular senescence. Biological sex represents a critical determinant in the prevalence and severity of musculoskeletal disorders; women exhibit a higher incidence and more rapid progression of OA or osteoporosis, particularly following the loss of estrogenic protection during menopause [53, 54]. While direct investigations into sex‐specific retrotransposon activation in joint tissues are currently limited, emerging evidence in other contexts suggests that sex hormones can modulate the epigenetic machinery that silences transposable elements [55, 56, 57]. Ultimately, targeting the dormant viral genome offers a paradigm‐shifting approach to preserving musculoskeletal health in an aging population.

## 7 Conclusion

Retrotransposons, particularly ERVs, act as critical sentinels of genomic stability, and their reactivation marks a pivotal tipping point in the pathogenesis of musculoskeletal diseases. ERVs remain a novel and largely underexplored axis in bone biology. Significant questions persist regarding the specific ERV families involved, the precise interplay between sex‐dimorphic immune responses and retrotransposon activation, and the window of opportunity for intervention. Current research has only begun to uncover the link between epigenetic dysregulation and viral mimicry in bone and joint tissues. Further mechanistic studies are required to define which specific ERV families are reactivated in human diseases and how they interact with host immune pathways. Importantly, current findings suggest that targeting ERV activation, either through antiretroviral drugs or epigenetic modulators, represents a promising and novel therapeutic strategy for treating age‐related musculoskeletal diseases.

## Author Contributions

Ye Liu drafted the manuscript; H. Josh Jang and Tao Yang revised and edited the manuscript.

## Funding

This work was supported by NIH funds (R01AG083568 and R01AG061086).

## Conflicts of Interest

The authors declare no conflicts of interest.

## Data Availability

Data sharing is not applicable to this article as no new data were created or analyzed in this study.
